# Statement of consensus on Family Medicine in Africa

**DOI:** 10.4102/phcfm.v2i1.151

**Published:** 2010-03-12

**Authors:** Robert (Bob) Mash, Steve Reid

**Affiliations:** 1 Division of Family Medicine and Primary Care, Stellenbosch University, Tygerberg campus, South Africa; 2 Faculty of Health Sciences, University of Cape Town, South Africa

## Abstract

Family Medicine is an emerging speciality in sub-Saharan Africa and yet potential interest in the contribution of Family Medicine to health, primary care and district health services is limited by the lack of a regional definition. Governments, health departments and academic institutions would benefit from a clearer understanding of Family Medicine in an African context.

The 2nd African Regional WONCA (World Organisation of Family Doctors) Conference, held in Rustenberg, South Africa in October 2009, engaged participants from sub-Saharan Africa in the development of a consensus statement on Family Medicine. The consensus statement agreed to by the conference defined the contribution of Family Medicine to equity, quality and primary health care within an African context, as well as the role and training requirements of the family physician. Particular attention was given to the contribution of women in Family Medicine.

## INTRODUCTION

### Points of recognition

The participants at the 2nd African Regional WONCA (World Organisation of Family Doctors) Conference that was held in Rustenburg, South Africa from 25 to 28 October 2009 noted the following: 

- the universal and recognised human right to health
- the gross inequalities and disparities in health status and health care within Africa, as well as between Africa and the rest of the world
- the limitation of resources for health care in Africa
- the strength of extended family values, interdependence and community accountability and the continuity between individual, family and community systems in Africa 
- the variety of perceptions and beliefs with regard to health care that stem from Africa’s cultural, religious and ethnic diversity
- gender inequality and the status of girls’ and women’s health directly influence the health of families and communities; gender is an important social determinant of health
- the Millennium Development Goal targets to be achieved by 2015
- the significant value of Family Medicine to the quality and equity of health services in many countries around the world
- the support of WONCA, governments, academic institutions, non-governmental organisations, donors and other stakeholders in Family Medicine.

The recognition of these points led to the development of an eight-tiered statement of consensus on the role of Family Medicine in Africa.

## STATEMENT OF CONSENSUS

### 1. The contribution of Family Medicine to equity in health care

1.1 Family Medicine, a core contributor to primary health care, is critical to the achievement of equitable health outcomes for all.

1.2 All people should have equal access to health care. In particular, access should not be limited by the inability to pay, or the lack of health care providers or facilities. Care should be focused on people and not specific diseases, as is the situation with regard to the funding of vertical health programmes. Access should not be limited by geography, culture, gender, religion, administration, policy or disability.

1.3 In order to deliver better health outcomes for all, the principles of Family Medicine should be shared by the whole primary health care team. They include the family physician, the general practitioner, the clinical nurse practitioner, the midwife, mid-level workers (including clinical/medical officers and assistants) and community-based health workers.

1.4 Training institutions should be socially accountable in adequately preparing health workers with the correct knowledge, attitudes and competencies, so that they are able to play their part in the primary care team.

1.5 Family Medicine should advocate for social and health policies that promote equitable health care. For example, health funding should be allocated according to the health needs of the population and incentives should be provided to attract health workers to underserved communities in order to improve the quality of care.

1.6 Family Medicine should advocate for the resolution of conflicts and promote peace as a fundamental prerequisite for the provision of equitable health care.

1.7 Family Medicine should practice cost-effective care in order to attain maximum value from limited resources. 

1.8 Family Medicine should contribute to quality care that is not threatened by commercialisation, or by weak services in the public sector. 

1.9 Family Medicine should empower people in communities to tackle the social determinants of ill-health.

### 2. Family Medicine and primary health care 

2.1 The concept of comprehensive primary health care and the principles of Family Medicine overlap considerably and should be considered together.

2.2 The World Health Organization resolutions on strong decentralised district health systems need to be implemented in Africa.

2.3 The clinical practice of Family Medicine is integral to the district health system and includes care at community, clinic, health centre and hospital levels. 

2.4 Family Medicine should strive to create an integrated system of health care that includes private, faith-based and traditional health care providers and supports inter-sectoral collaboration and active community participation.

2.5 Family Medicine should be delivered by a team with the appropriate skills mix and strong teamwork.

2.6 Family Medicine plays a clear gate-keeping role in referring patients to the rest of the health care system.

2.7 Public–private partnerships that are socially accountable (e.g. not for profit) have the potential to improve health care outcomes in terms of service delivery, teaching and research. 

2.8 All governments in Africa should create viable frameworks to support health for all through the inclusion of family physicians in primary health care teams.

### 3. The role of the family physician in Africa

3.1 In an African context, the family physician is a clinical leader and consultant in the primary health care team, ensuring primary, continuing, comprehensive, holistic and personalised care of high quality to individuals, families and communities.

3.2 The family physician in Africa operates according to the principles of comprehensive person-centred care, with a family and community orientation, responding to undifferentiated illness and acting as a consultant to the primary health care team.

3.3 The role of the family physician in Africa involves a comprehensive set of skills adapted to the circumstances, local needs, available resources, facilities and the competency and limitations of the practitioner.

3.4 The family physician has a commitment and responsibility to a defined population to whom they are accountable through its representative structure.

3.5 The family physician’s role requires close collaboration and teamwork with other members of the primary health care team, especially in the light of specific challenges, such as the insufficient numbers of health care workers.

3.6 The limited human, financial and material resources which exist necessitate skills appropriate to the situation. The family physician’s responsibility as consultant and gate-keeper encompasses the economic, effective and efficient use of available resources (human, financial and informational), as well as the ability to prioritise. 

3.7 The family physician is also a life-long scholar, which includes a commitment to life-long learning, research and audit, and a responsibility for the continuing education of the primary health care team and community.

3.8 The family physician is an interdisciplinary player, with a pivotal role in the coordination of the primary health care team, including leadership in clinical governance and patient referrals.

3.9 Cultural competency – in relation to language, gender, traditions and religious beliefs – is an essential attribute.

3.10 The family physician must play an advocacy role, both through daily example and through their institutions, by actively identifying with, and advocating for, the poor and marginalised. 

3.11 The family physician should generate social and managerial accountability and transparency in terms of effective and efficient health care delivery.

3.12 Family physicians have a responsibility for health resource and service management based on their clinical understanding and should have direct access to District Health Management Teams.

3.13 The family physician may focus on various areas of special interest at different times in their career. At the same time, they must remain competent across a broad scope of practice as a generalist.

### 4. The African context and community

4.1 Family Medicine is community-based and must be embedded in all settings within the District Health System.

4.2 Family Medicine practice should always be community-oriented and context-specific. The starting point is a defined community. Family physicians’ care for individuals is informed by their community orientation, with their community role arising out of their clinical role. Community-oriented primary care is one tool that family physicians can use to make the link between individual and community care. 

4.3 Family physicians must consistently be aware of, and continue to address, the priority health needs of the community. 

4.4 Family physicians are committed to improving the health of all the people in the communities they serve, not just those who are able to access care. They must be recognised and rewarded for the quality of care and the improved health of all members of the community. 

4.5 Context is critical for training and should, to a large extent, determine the curriculum. Family physicians must be provided with the appropriate contextual training that will enable them to identify and address community needs.

4.6 Family physicians must understand, be sensitive to, and work within, the culture of the community, as well as the ongoing changes to this culture. Family physicians must respect the family and community values of the people they serve, encouraging those that support positive health outcomes. 

4.7 Family physicians must work with, and in, teams that are defined by the context, as team members and not only as leaders. They play a supportive and consultative role in the primary health care team.

4.8 Appropriate resources must be allocated for community-based training, research and practice. 

### 5. Quality of Family Medicine practice in Africa

5.1. Family Medicine regards good clinical governance and quality of primary health care as fundamental to the profession. This will be achieved through sound training and maintained by continuous, peer-reviewed, medical education that is formally accredited.

5.2. Family physicians should perform regular reviews and audits to enable reflection on their work and service, building on strengths and correcting weaknesses in the primary health care system on a regular basis.

5.3. Appropriate tools and systems for the evaluation of Family Medicine practice need to be developed, and indicators defined to benchmark practice in Africa. This should be based on key domains of quality, which include cost effectiveness, safety, equity, continuity of care and patient satisfaction. 

5.4. It is recognised that maintaining the quality of services requires well-motivated family physicians working within an environment that provides adequate human and physical resources.

5.5. Quality service must be rewarded.

5.6. The practice of Family Medicine should, as far as possible, be evidence-based.

### 6. Family physician training in sub-Saharan Africa

6.1. Training should take place in all the District Health Services where teachers of Family Medicine function, with in-patient, out-patient, and outreach programmes.

6.2. Both full-time residential and part-time training programmes may be necessary to maximise training opportunities. 

6.3. Teaching sites must be of sufficient size, with a ‘critical mass’ of trainers to create teaching and practice centres of excellence. 

6.4. Experience in the management of chronic disease and undifferentiated illness should be part of the training throughout the programme.

6.5. Research must be included in all graduate programmes. 

6.6. Training should include cross-cutting themes of specific relevance to Family Medicine, such as communication and consultation skills, reflective practice, holistic care, health systems management, and how to teach.

6.7. Training should be outcome- and competency-based.

6.8. Training should have a strong academic (university-based) foundation. 

6.9. The principles of Family Medicine should be introduced early in undergraduate medical training and continue throughout the training. 

6.10. Internship programmes should include rotations with exposure to family physicians and Family Medicine registrars. 

6.11. Length of postgraduate Family Medicine training should be sufficient to teach core competencies and prepare for life-long learning. The length of the program must take into account the local postgraduate university requirements.

6.12. All relevant stakeholders (e.g. Ministries of Health and Education, Medical Councils, and professional organisations) should ideally be involved from the start of Family Medicine training programmes. 

6.13. Trained and qualified family physicians should have consultant status and be remunerated at the same level as other specialists.

6.14. Family physicians should be locally produced and of international standard.

6.15. The family physician should be involved in the training of medical personnel in the public and the private sectors. Private-sector facilities may form part of the training sites for undergraduate students. 

6.16. The family physician should collaborate with general practitioners in the development of clinical guidelines. 

6.17. Training of family physicians should include an understanding of traditional health practices and integrative medicine. 

6.18. Training should have equivalent length to other specialities, especially with the need for procedural skills in an African context. 

6.19. Training should allow registrarship in Family Medicine during community service.

6.20. Training should be outcome-based and the location of training will depend on the learning opportunities offered.

6.21. The rotation of a specific registrar must depend on their prior skills and competency.

### 7. Women in Family Medicine in Africa

7.1. WONCA Africa Region commits to uphold: the Hamilton Equity regulations, the Ten Steps to Gender Equity, and the by-law changes pertaining to equity within the governance and activities of the organisation, as proposed by the WONCA Working Party on Women and Family Medicine – recommended by the WONCA Executive, and due to be approved by WONCA World Council. 

7.2. All family physicians need to be advocates for gender equality; female practitioners should act as role models for girls in general, as well as for female patients and colleagues. 

7.3. The Life Care Model adopted by WONCA internationally should be promoted in Africa (see Table 1). 

7.4. Leadership amongst female family physicians should be promoted and supported at all levels, leading to proportional representation in leadership positions within the next 5 years. 

7.5. A network of female family physicians should be developed. 

7.6. Gender-based research and empowerment of female researchers should be promoted.

**TABLE 1:**
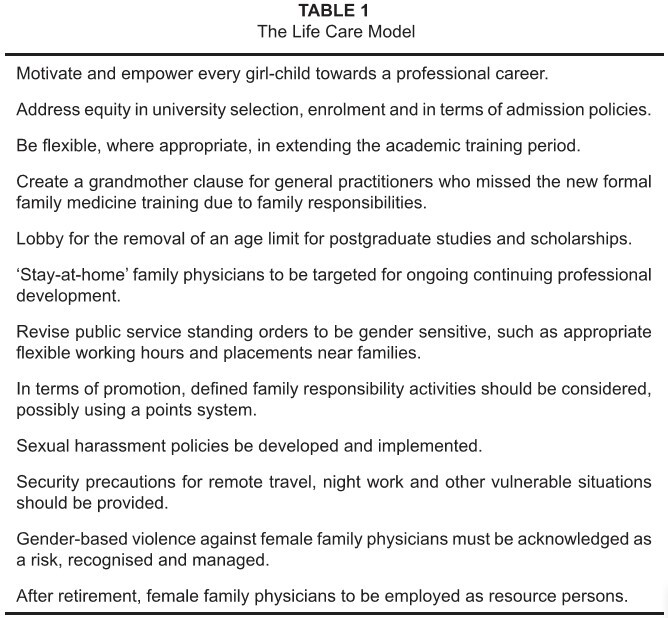
The Life Care Model

### 8. Nomenclature

8.1. The term ‘family doctor’ should be understood as referring to the following: family physician, general practitioner, or medical officer.

8.2. A family physician has postgraduate training in Family Medicine. The term ‘family physician’ should be the nomenclature in Africa. 

8.3. A medical officer is a generalist in the public sector, without postgraduate training.

## DEVELOPMENT PROCESS

The consensus statement was the result of a participatory process which culminated in an African Regional WONCA conference. In the year preceding the conference, a number of initiatives prepared the ground for the development of a consensus statement. These initiatives included:

- May 2008: Publication of a study, ‘Exploring the key principles of Family Medicine in sub-Saharan Africa: International Delphi consensus process’, that enabled an expert panel to reach consensus on the key principles of Family Medicine. ^1^
- Primafamed Conference, Kampala, Uganda, 17–22 November 2008: ‘Improving the quality of Family Medicine training in sub-Saharan Africa’. This conference enabled dialogue between family physicians in sub-Saharan Africa. The initial conference themes and plans for discussion papers emerged from the dialogue at this conference.
- Discussion papers on all the key themes (except Women in Family Medicine, which was a late submission) were written and published in the WONCA Regional Conference brochure, as well as on the WONCA website. These discussion papers were designed to stimulate initial thinking and debate at the conference.
- The WONCA Working Party on Women in Family Practice held a pre-conference workshop during which their recommendations were generated. 
- The conference was attended by 294 participants from various African countries and elsewhere (see acknowledgements). The conference was designed around eight themes that had been previously identified at the regional Primafamed Conference and by the scientific committee.

These themes were:

1. African context and community
2. Primary health care and health systems
3. Training in Family Medicine
4. Ensuring quality of care
5. Role and scope of practice of the family practitioner 
6. Equity and Family Medicine
7. Private practice, faith-based hospitals and private–public partnerships
8. African family values and women in Family Medicine.

Key note speakers, in addition to the discussion papers on each theme, were included in the conference programme to stimulate dialogue. The conference itself was designed as a participatory process, whereby all the participants signed up to a small group of their choice which focused on one of the conference theme areas. Each small group consisting of a maximum of 20 people, met twice over a total period of 4 hours and, after deliberation, created a 250 word statement on their theme, to be included as a paragraph in the final conference statement. Each group was led by a facilitator and utilised a modified nominal group technique to prioritise the group’s recommendations. Facilitators who led groups on the same theme gave feedback to one another halfway through the small group process and, at the end, combined their recommendations into one joint paragraph.

The final paragraphs on each theme were presented to a plenary session and discussed by all the conference participants. Participants in the plenary session had the opportunity to suggest amendments and additions to the wording of the paragraphs. Following this, the revised paragraphs were voted on, point by point by a show of hands in the final plenary session. If the total number of those who abstained, or those who disagreed with a point in the statement was more than 25% of those present in the final plenary session, then the point was excluded from the final consensus statement (see Table 2). The reason for rejection was not clarified, but, in addition to disagreement with the concept, may also have been because of poor wording or duplication elsewhere.

**TABLE 2:**
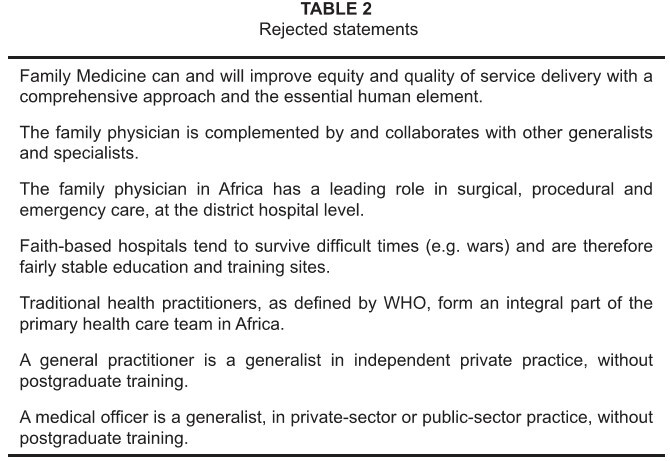
Rejected statements
